# Evaluation of thromboembolic event, basic coagulation parameters, and associated factors in patients with colorectal cancer: a multicenter study

**DOI:** 10.3389/fonc.2023.1143122

**Published:** 2023-05-02

**Authors:** Fitalew Tadele Admasu, Tadesse Asmamaw Dejenie, Gashaw Walle Ayehu, Edget Abebe Zewde, Gashaw Dessie, Dagnew Getnet Adugna, Engidaw Fentahun Enyew, Zeleke Geto, Endeshaw Chekol Abebe

**Affiliations:** ^1^ Department of Biochemistry, School of Medicine, College of Medicine and Health Sciences, Debre Tabor University, Debre Tabor, Ethiopia; ^2^ Department of Biochemistry, School of Medicine, College of Health Sciences and Medicine, Gondar University, Gondar, Ethiopia; ^3^ Department of Anatomy, School of Medicine, College of Medicine and Health Science, Gondar University, Gondar, Ethiopia; ^4^ Department of Biomedical Sciences, School of Medicine, College of Health Sciences, Wello University, Wello, Ethiopia

**Keywords:** coagulopathy, thromboembolic event, basic coagulation profiles, colorectal cancer, Ethiopia

## Abstract

**Background:**

Patients with colorectal cancer are at an increased risk of hemostatic disturbances, and recent studies have shown that coagulation disorders could be the first sign of malignancy. Although coagulopathy is a significant cause of cancer-related death and disability, it is usually underestimated, and there has been no recent scientific evidence regarding the exact burden and its specific determinants. Moreover, the public health importance of the risk of coagulopathy among patients with colorectal polyps has not been addressed.

**Materials and methods:**

An institution-based comparative cross-sectional study was conducted on a total of 500 study participants (250 colorectal cancer patients, 150 colorectal polyp patients, and 100 controls) from January to December 2022. Venous blood was collected for basic coagulation and platelet analysis. Descriptive statistics and non-parametric tests (Kruskal–Wallis and Dunn–Bonferroni pairwise comparisons) were used to compare study parameters among the groups. The test results were expressed as medians and interquartile ranges. Binary logistic regressions were fitted, and statistical significance was declared at a *p*-value of less than 0.05, with 95% CI.

**Results:**

The prevalence of coagulopathy among colorectal cancer patients was 198 (79.2%; 95% CI: 73.86, 83.64), while the prevalence was 76 (50.7%; 95% CI: 45.66, 54.34) among colorectal polyp patients. From the final model, age between 61 and 70 (AOR = 3.13: 95% CI: 1.03, 6.94), age > 70 years (AOR = 2.73: 95% CI: 1.08, 4.71), hypertension (AOR = 6.8: 95% CI: 1.07, 14.1), larger tumor size (AOR = 3.31: 95% CI: 1.11, 6.74), metastatic cancer (AOR = 5.8: 95% CI: 1.1, 14.7), and BMI ≥30 kg/m^2^ (AOR = 3.8: 95% CI: 2.3, 4.8) were positively associated with coagulopathy.

**Conclusion:**

This study showed that coagulopathy is a major public health concern among patients with colorectal cancer. Therefore, existing oncology care efforts should be strengthened to prevent coagulopathy among patients with colorectal cancer. Moreover, patients with colorectal polyps should receive more attention.

## 1 Introduction

Colorectal cancer (CRC) is a significant cause of morbidity and mortality worldwide, and it is especially on the rise in sub-Saharan African countries (1). Worldwide, approximately 35 million people die due to non-communicable diseases like cancer, of which approximately 80% of deaths occur in developing countries (2, 3). CRC, which comprises approximately 10.2% of gastrointestinal (GI) cancers, is relatively prevalent with lethal groups of malignancy arising from the inner lining of the colon or rectum (4). Globally, GI cancers account for one in four cancer cases and one in three cancer deaths, and in recent times, although the overall prevalence of GI cancer has decreased, CRC incidence has increased globally, including in formerly low-incidence regions (5). According to WHO, CRC is the third most commonly diagnosed cancer in men and the second in women, and the lifetime risk of developing CRC is also high, estimated to be 1 in 23 (4.3%) for men and 1 in 25 (4.0%) for women (6). In the United States, CRC is the third leading cause of cancer-related deaths and the second most common cause of cancer-related deaths. In 2022, it was estimated to cause approximately 52,580 deaths, and in younger people, deaths from CRC have increased by 1% per year since 2008 (7, 8). CRC is one of the most common causes of morbidity and mortality in developing countries, and its occurrence is higher than that in other European, American, and Chinese countries (9, 10). In Africa, mortality from CRC has increased over time (3) due to the increased prevalence of risk factors such as growth and aging of the population, sedentary lifestyle, lack of modern treatment system, late presentation, increased comorbid illness, and poor awareness (11, 12).

Acute and chronic cancer-related complications that arise either as an initial malignancy manifestation or due to its progression are often the main cause of cancer-related mortality and hospital admission. These complications influence the prognosis and timing of cancer diagnosis, as well as the timing and receipt of treatment outcomes (13).

Cancer patients are usually associated with an increased risk of hemostatic disturbances and thromboembolic events (6, 14) and recent studies have shown that coagulation disorders could be the first sign of malignancy, and coagulation and fibrinolysis indicators are deranged in the blood-supplying tumors and peripheral blood of cancer patients, suggesting that cancer can directly affect the coagulation and fibrinolytic systems (15). Among CRC patients, clinical and subclinical changes in the coagulation and fibrinolysis systems, thrombotic disease, and disseminated intravascular coagulation are particularly common (16). Since it was first identified in the early 19th century by Professor Armand Trousseau [Trousseau’s syndrome (TS)] (17), thromboembolic events have become an important public health burden for cancer patients and are associated with a complicated surgery, hospitalizations, and systemic therapies (18). Among patients with CRC, thromboembolic complications are becoming the second leading cause of death by interrupting or delaying essential cancer treatments, often with increased healthcare resource utilization (19, 20).

Recent international studies have reported that multiple risk factors, including patient-related (advanced age, race, sex, comorbidities, obesity, and history of thrombosis), cancer-related (primary tumor site, disease stage/grade, cancer histology type, and duration since initial diagnosis), and cancer treatment-related (chemotherapy, radiotherapy, surgery, anti-angiogenic agents, hormonal therapy, and transfusions) factors are associated with an increased risk of developing coagulopathy (21, 22). The overall prevalence and mortality rates of CRC are higher in men due to a number of biological and gender-related behavioral factors like high consumption of red and processed meat, high rate of alcohol consumption, smoking, higher risk of visceral obesity, and the protective role of estrogen hormone in women; however, women were found to have more aggressive forms of the cancer (23). To avoid cancer-related complications, such as coagulopathy, minimize mortality, and enhance patient quality of life, it is critical to recognize and manage risk factors early. In Ethiopia, despite the increased incidence and prevalence of CRC and its complications, the relatively high incidence of CRC mortality, and the high rate of delay to diagnosis, CRC receives a relatively low public health priority due to low public awareness, scarce financial resources, weak healthcare systems, a shortage of oncologists, and an already overburdened economy (24). Evidence also indicated that the Ethiopian public lacks a comprehensive understanding of CRC and its risk factors, warning signs and symptoms, and early and late complications (25).

However, data on the prevalence and determinants of coagulopathy among patients with CRC in Ethiopia are limited. Moreover, documented data about the prevalence and associated factors of coagulopathy among CRC patients are important to understand the disease burden, identify high-risk groups, and develop effective preventive strategies. From this perspective, this study aimed to assess the prevalence of thromboembolic events and the prevalence and determinants of coagulopathy among adult CRC patients visiting the oncology units of public referral hospitals in Addis Ababa, Ethiopia.

## 2 Materials and methods

### 2.1 Study design, setting, and period

An institution-based comparative cross-sectional study was conducted from January to December 2022 at oncology centers in referral hospitals of Addis Ababa, Ethiopia, namely, Tikur Anbessa Specialized Hospital, Zewditu Memorial Hospital, and St. Paul Specialized Hospital. Currently, these hospitals provide basic cancer diagnosis, follow-up, chronic care, chemo/radiotherapy, surgery, and other services to almost all Ethiopian clients.

### 2.2 Study participants, sample size, and sampling techniques

The study was conducted with 500 participants. In this study, all adult participants (≥18 years old) of both sexes were included. The study participants were divided into three groups: Group I comprised 250 histopathologically confirmed CRC patients, diagnosed and on chronic follow-up; CRC patients who were admitted for anticancer treatment (chemotherapy/surgery); and CRC patients admitted due to any cancer-related complication management but who did not receive any type of anticancer treatment at the selected hospitals during the data collection period. To analyze and compare the evaluated basic coagulation parameters, in addition to CRC patients, our study also recruited 150 histopathologically confirmed (colonoscopy or flexible sigmoidoscopy) colorectal polyp (CRP) patients who visited the selected hospitals or were on chronic follow-ups, and who did not receive any type of treatment during the data collection period as group II and 100 apparently healthy volunteers as the third group (group III), which included apparently healthy adults who visited the selected study areas for any reason (clinical and administrative staff members, patient attendants) during the study period. For all healthy controls, after screening (detailed medical record review and physical examination) for any acute febrile illness and chronic disease, including known cancer, CT scan imaging was performed for any lesion. Finally, individuals who fulfilled the selection criteria and had no lesions were included (sex and age range matched).

### 2.3 Inclusion and exclusion criteria

All adult CRC and CRP patients whose age was older than 18 years and visiting the selected hospitals during the study period (from January 2022 to December 2022) were included in the study.

Starting from 1 January 2022, all consecutive patients (CRC and CRP) were evaluated on the first day of diagnosis and/or hospitalization, and those fulfilling the eligibility criteria were included. Study participants on chronic anticoagulation therapy, those with a history of thromboembolic events in the past 3 months, and pregnant women were excluded from the study for all groups.

### 2.4 Data collection procedures

#### 2.4.1 Sociodemographic and clinical data collection

To collect baseline information on study participants’ sociodemographic characteristics and related information, an interviewer-based pretested and structured questionnaire was used. Clinical and histopathological data [medical conditions/comorbidities, HIV/AIDS status, primary tumor location (colon, rectal), tumor size, sites of metastases, risk factors for coagulopathy, and BMI] were abstracted from the patient’s medical chart by medical record review using a structured checklist with a physical examination. The tools were prepared by reviewing many similar international studies with some modifications in the local context, but most were developed by researchers after consulting oncologists and hematologists. The questionnaire was first prepared in English and then translated into Amharic, and the final tool was prepared in English after retranslating the Amharic version for consistency purposes. Pretests were performed on 5% of the total sample size, and modifications were made accordingly.

#### 2.4.2 Blood sample collection and laboratory analysis procedure

Before surgery or any other treatment modality, the study participants were well-informed and made aware of the aim of the study. After obtaining written informed consent, blood samples and responses to the questionnaires were collected by qualified laboratory personnel following standard operating procedures (SOPs). A total of 500 fasting blood samples (250 CRC patients, 150 CRP patients, and 100 controls) with a volume of approximately 8 ml were collected and dispensed into a test tube containing 0.3 ml of 3.2% trisodium citrate, and platelet-poor plasma (PPP) was obtained after centrifugation at 1,500 *g* for 15 min. Then, using PPP, basic coagulation parameters PT (prothrombin time), APTT (activated partial thromboplastin clotting time), and INR (international normalized ratio) were analyzed using a HUMACLOT DUE PLUS coagulation analyzer (Wiesbaden, Germany). During the analysis, the analyzer read the clotting time of APTT and displayed the result in seconds, and the time taken for clot formation (in seconds) was recorded for the PT measurement. The INR was calculated and displayed from the PT output. For platelet count, approximately 3 ml of whole blood was analyzed using the SYSMEX K-21N automated hematology analyzer (Sysmex Corporation, Kobe, Japan). All laboratory analyses were conducted following SOPs and the manufacturers’ recommendations. All laboratory analyses were conducted on the same day as blood sample collection.

#### 2.4.3 Thromboembolic and bleeding events

The study participants (CRC and CRP patients) were also assessed for the development of thromboembolic and bleeding events. For all included CRC patients, contrast-enhanced CT was performed, and a diagnosis of a thromboembolic event was made based on the description by the original reporting radiologist. We also included patients with CRC who developed all types of thromboembolic events detected by CT or additional imaging tests within 1 month of admission. Upon reviewing the medical records of patients with CRC on chronic follow-up, radiologic imaging was repeated if the imaging was performed before a month. The same imaging procedure was performed for the CRP patient groups.

Bleeding events were assessed according to the International Society on Thrombosis and Hemostasis definitions of major bleeding and clinically relevant non-major bleeding.

#### 2.4.4 Operational definitions

Coagulopathy was considered an abnormality in one of the basic coagulation parameters assessed: prolonged PT and/or APTT values, thrombocytopenia, abnormally high PT/international normalized ratio (INR), or APTT.

Normal time for PT: The normal time for PT was considered between 10 and 14 s.

Normal time for APTT: The normal APTT time was considered 24–36 s.

Abnormal high INR: The normal INR was considered 0.8–1.2.

Normal platelet count: Platelet count between 150,000 and 400,000/µl.

### 2.5 Data processing and statistical analysis

Before analysis, the data were checked for completeness and internal consistency, coded and entered into Epidata version 4.6, and analyzed using STATA software version 16. Descriptive statistics, such as percentages, mean, median, IQR, and standard deviations, were used to present the sociodemographic and clinical characteristics of the study participants. Non-parametric Kruskal–Wallis tests followed by Dunn–Bonferroni pairwise comparison tests were used to compare the median (IQR) values of the different serum parameters between the case and control groups. Binary logistic regression analysis was used to examine independent variables associated with coagulopathy. Bivariate logistic regression was used to rank the relative importance of each independent variable with the outcome variables using odds ratios. Variables with a *p*-value of less than 0.25 in the bivariate analysis were chosen for multiple logistic regression analysis. Finally, AOR with 95% confidence intervals (CIs) was used, and *p*-values less than 0.05 were used to determine statistically significant factors.

### 2.6 Data quality assurance

Before actual data collection, training and discussion with the data collectors, laboratory technicians, and supervisors were undertaken regarding the content of the questionnaires, data collection techniques, and measurement procedures. Before data collection, a pretest was performed on 5% of the sample size to determine the effectiveness and consistency of the questionnaire under the direct supervision of an oncologist, after the supervisors and principal investigator checked them daily for accuracy, consistency, and completeness. Blood samples were collected using aseptic techniques and standard operating procedures. The kit was free of contamination and checked for consistency. All laboratory procedures were handled by professional laboratory technologists, and the results were checked daily for completeness by an immediate supervisor.

This cross-sectional study was appraised according to the Strengthening The Reporting of Observational Studies in Epidemiology (STROBE) (Appendix A) (26).

## 3 Results

### 3.1 Sociodemographic characteristics of study participants

A total of 500 adult participants participated in this study with a 100% response rate. The study participants were in three groups: 250 CRC patients, 150 CRP patients, and 100 healthy controls. The median ages of the CRC patients, CRP patients, and control groups were 42.5, 57.0, and 50.0 years, respectively. In the study, only 4 (1.6%), 1 (0.7%), and 1 (1.0%) of study participants had a family history of CRC for the respective CRC, CRP, and control groups (**Table 1**).

**Table 1:** Sociodemographic characteristics of study participants at the referral hospitals of Addis Ababa, Ethiopia, 2022.

Variables	Categories	CRC (N = 250) N (%)	CRP (N = 150) N (%)	Control group (N = 100) N (%)
Age (years)	≤50 51–60 61–70 >71	149 (59.6) 39 (15.6) 42 (16.8) 20 (8.0)	21 (14.0) 30 (20.0) 41 (27.4) 58 (38.6)	31 (23.0) 26 (26.0) 37 (37.0) 6 (6.0)
Gender	Male Female	157 (62.8) 93 (37.2)	97 (64.7) 53 (35.3)	59 (59.0) 41 (41.0)
Residence	Urban Rural	98 (39.2) 152 (60.8)	68 (45.3) 82 (54.7)	60 (60.0) 40 (40.0)
Marital status	Single Married Widowed	90 (36.0) 145 (58.0) 15 (6.0)	30 (60.0) 13 (26.0) 7 (14.0)	17 (17.0) 73 (73.0) 10 (10.0)
Educational status	Illiterate Primary school Secondary school College/University	33 (13.2) 62 (24.8) 41 (16.4) 114 (45.6)	28 (18.7) 30 (20.0) 30 (20.0) 50 (33.3)	7 (7.0) 7 (7.0) 10 (10.0) 76 (76.0)
Religion	Orthodox Protestant Muslim Other	92 (37.0) 85 (34.0) 70 (28.0) 3 (1.2)	81 (54.0) 33 (22.0) 35 (23.3) 1 (0.7)	46 (46.0) 29 (29.0) 25 (25.0) 0 (0.0)
Alcohol consumption	Yes no	17 (6.8) 233 (93.2)	14 (9.3) 136 (90.7)	7 (7.0) 93 (93.0)
Smoking	Yes no	18 (7.2) 232 (92.8)	7 (4.7) 143 (95.3)	2 (2.0) 98 (98.0)
Family history of colorectal cancer	Yes No Unspecified	4 (1.6) 102 (40.8) 144 (57.6)	1 (0.7) 50 (33.3) 99 (66.0)	1 (1.0) 50 (50.0) 49 (49.0)
Family history of colorectal polyp	Yes No Unspecified	2 (0.8) 52 (20.8) 196 (78.4)	1 (0.7) 50 (33.3) 99 (66.0)	1 (0.01.0) 50 (50.0) 49 (49.0)
Family history of bleeding	Yes No Unspecified	2 (0.8) 52 (20.8) 196 (78.4)	1 (0.7) 50 (33.3) 99 (66.0)	1 (0.01) 50 (50.0) 49 (49.0)
Physical exercise habits	Never Once a week 2–3 times a week 4–6 times a week	153 (61.2) 78 (31.2) 14 (5.6) 5 (2.0)	81 (54.0) 22 (14.7) 36 (24.0) 11 (7.3)	59 (59.0) 22 (22.0) 13 (13.0) 6 (6.0)
Body mass index (kg/m^2^)	Underweight (<18.5) Normal weight (18.5–24.9) Overweight (25–29.9) Obese (≥30)	80 (32.0) 130 (52.0) 34 (13.6) 6 (2.4)	10 (6.7) 92 (61.3) 41 (27.3) 7 (4.7)	5 (5.0) 64 (64.0) 24 (24.0) 7 (7.0)
Hypertension	Yes No	19 (7.6) 231 (92.4)	14 (9.3) 136 (90.7)	6 (6.0) 94 (94.0)
Diabetes mellitus	Yes No	2 (0.8) 248 (99.2)	0 (0.0) 150 (100.0)	1 (1.0) 99 (99.0)
Cardiac disease	Yes No	2 (0.8) 248 (99.2)	0 (0.0) 150 (100.0)	1 (1.0) 99 (99.0)
HIV/AIDS	Yes No	13 (5.2) 237 (94.8)	17 (11.3) 133 (88.6)	23 (23.0) 77 (77.0)

CRC, colorectal cancer; CRP, colorectal polyp.

### 3.2 Histological, pathological, and clinical characteristics of study participants

Out of the 250 CRC patients, more than half (148, 59.2%) had a tumor located in the colon. Histopathological examination showed that adenocarcinoma (199, 79.6%) and mucinous adenocarcinoma (34, 13.6%) were the common histologic types, and 116 (46.4%) and 47 (18.8%) were moderately and poorly differentiated, respectively. Among the 150 CRP patients who participated, 122 (81.3%) had only one polyp, and histological examination revealed that the neoplastic histological type was dominant (103, 68.6.3%), of which 50 (48.5%) were tubulovillous adenomas and 32 (31.1%) were tubular adenomas (**Table 2**).

**Table 2:** Histological, pathological, and clinical characteristics of study participants at referral hospitals of Addis Ababa, Ethiopia, 2022.

Variables	Categories	CRC N = 250 N (%)	CRP N = 150 N (%)
Primary tumor site	Colon Rectum	148 (59.2) 102 (40.8)	108 (72.0) 42 (28.0)
Tumor histopathology	Adenocarcinoma Mucinous adenocarcinoma Signet ring cell adenocarcinoma Unspecified	199 (79.6) 34 (13.6) 12 (4.8) 5 (2.0)	NA
Tumor grade	Well differentiated Moderately differentiated Poorly differentiated Undifferentiated	54 (21.6) 116 (46.4) 47 (18.8) 33 (13.2)	NA
Tumor size (cm)	0–2 2–5 5–10 ≥10	28 (11.2) 136 (54.4) 77 (30.8) 9 (3.6)	NA
Distant metastasis	Yes No	124 (49.6) 126 (50.4)	NA
Lymph node involvement	Node negative Node positive Node unknown	59 (23.6) 175 (70.0) 16 (6.4)	NA
Lymph vascular invasion	Yes No Unspecified	6 (2.4) 152 (60.8) 92 (36.8)	NA
CEA level	Not elevated (≤5 ng/ml) Elevated (>5 ng/ml) Unspecified	110 (44.0) 131 (52.4) 9 (3.6)	NA
Duration since diagnosed (years)	<1.5 years 1.5–3 years >3 years	150 (60.0) 62 (24.8) 38 (15.2)	72 (48.0) 41 (27.3) 37 (24.7)

CRC, colorectal cancer; CRP, colorectal polyp; CEA, carcinoembryonic antigen; NA, not applicable.

### 3.3 Basic coagulation and platelet profiles of study participants

In this study, compared to apparently healthy and CRP patients, PT, INR, and APTT were prolonged in most CRC patients, of whom 190 (76%), 178 (71.2%), and 107 (42.8%) had prolonged PT, INR, and APTT, respectively, and 65 (26%) CRC patients had thrombocytopenia (**Table 3**).

**Table 3:** Basic coagulation profiles and platelet parameters of study participants at referral hospitals of Addis Ababa, Ethiopia, 2022.

Variable	Categories	CRC (N=250) N (%)	CRP (N=250) N (%)	Control groups (N=250)N (%)	Reference range
PT(sec.)	Range	9.9-27.2	9.5-17.9	9.8-14.4	10s–14s
	Normal	60(24.0)	89(59.3)	90(90.0)	
	Prolonged	190 (76.0)	61 (40.7)	10 (10.0)	
INR	Range	0.92-2.17	0.79-1.47	0.75-1.15	0.8–1.2
	Normal	72(28.8)	92(61.3)	90(90.0)	
	Prolonged	178 (71.2)	58 (38.7)	10(10.0)	
APTT(sec.)	Range	21.7-72.7	28.7-49.2	27.3-43.4	24s–36s
	Normal	143(57.2)	113(75.3)	93(93.0)	
	Prolonged	107 (42.8)	37 (24.7)	7 (7.0)	
PLT (×103/ μl)	Range	74-635	94–434	139-251	150-400*10^3^/μl
	Low	65 (26.0)	12 (8.0)	1 (1.0)	
	Normal	180 (72.0)	138(92.0)	99 (99.0)	
	High	5(2.0)	0 (0.0)	0 (0.0)	

CRC, colorectal cancer; CRP, colorectal polyp; PT, prothrombin time; PTA, prothrombin time activity; INR, international normalized ratio; APTT, activated partial thromboplastin time; PLT, platelet count.

### 3.4 Commutative prevalence of coagulopathy among study participants

In this study, the overall prevalence of coagulopathy among CRC patients was 198 (79.2%; 95% CI: 73.86, 83.64), of whom 190 (76.0%) showed prolonged PT, 178 (71.2%) showed prolonged APPT, and 65 (26.0%) showed thrombocytopenia. Furthermore, the total prevalence of coagulopathy among CRP patients was 76 (50.7%; 95% CI: 45.66, 54.34). From them, the prevalence of prolonged PT was 40.7% (61/150), and 8.0% (12/150) had thrombocytopenia (**Table 3**).

### 3.5 Comparison of basic coagulation and platelet parameters among study groups

Non-parametric tests, Kruskal–Wallis followed by Dunn–Bonferroni pairwise comparison test, were used to compare the median and IQR values and the median and IQR differences in basic coagulation and platelet parameters between study groups. In the Kruskal–Wallis analysis, the median [IQR] PT, APTT, and INR showed statistically significant differences among the CRC, CRP, and healthy control groups (*p* < 0.001) (**Table 4**).

**Table 4:** Comparative analysis of basic coagulation and platelet parameters (Kruskal–Wallis followed by post hoc analysis using Dunn–Bonferroni correction) of study participants at referral hospitals of Addis Ababa, Ethiopia, 2022.

Variable	CRC (N = 250) Median [IQR]	CRP (N = 150) Median [IQR]		Healthy controls (N = 100) Median [IQR]		p-value
PT (s)	15.4 [2.96]	13.5 [2.69]		13.1 [2.46]		<0.001^*^
INR	1.37 [0.28]	1.18 [0.25]		1.11 [0.24]		<0.001*
APTT (s)	35.91 [11.7]	33.7 [10.30]		29.4 [5.22]		<0.001*
Platelet count	309 [106]	237 [85]		227 [83]		<0.001*
Multiple pairwise comparisons using Dunn–Bonferroni correction						
Parameters	CRC vs. Healthy control		CRC vs. CRP		CRP vs. Healthy control	
PT (s)	<0.001*		<0.001*		<0.001*	
INR	<0.001*		<0.001*		0.301	
APTT (s)	<0.001*		0.111		<0.001*	
Platelet count	<0.001*		<0.001*		0.271	

CRC, colorectal cancer; CRP, colorectal polyp; PT, prothrombin time; PTA, prothrombin time activity; INR, international normalized ratio; APTT, activated partial thromboplastin time; PLT, platelet count; IQR, interquartile range; *p < 0.005.

In multiple pairwise comparison analysis using Dunn–Bonferroni pairwise comparison, the median [IQR] values of PT showed a statistically significant difference across all study groups, while the median [IQR] values of INR and platelet count among CRC patients were significantly different compared to CRP and healthy controls (*p* < 0.001). The median [IQR] values of platelet count showed significant differences between the CRC and CRP patients (*p* < 0.001), but there was no significant difference between the CRP and healthy control groups (*p* ≥ 0.271) (**Table 4**).

### 3.6 Prevalence of thromboembolic and hemorrhagic events among thestudy participants

In this study, the overall prevalence of thromboembolic events among CRC patients was 17 (6.8%), of which 9 (52.9%) were in the form of pulmonary embolism, followed by deep venous thrombosis (3, 17.6%), and most of the pulmonary embolisms were asymptomatic segmental (7/9, 77.8%). Thromboembolic events were also observed in the examined CRP patients, with a cumulative prevalence of 6 (4%), and the majority (4/6, 66.7) were in the form of pulmonary embolism. Bleeding events were observed in 2 (0.8%) CRC patients (**Table 5**).

**Table 5:** Detailed characterization of thromboembolic and hemorrhagic events among the study participants at referral hospitals of Addis Ababa, Ethiopia, 2022.

Variable	Type of thromboembolic event	Categories	CRC (*n* = 250) *N* (%)	CRP (*n* = 150) *N* (%)
Type of thromboembolic event	Pulmonary embolism	Asymptomatic, segmental	7 (2.8)	4 (2.7)
		Symptomatic, segmental	2 (0.8)	0 (0.0)
		Heavily symptomatic and/or bilateral	1 (0.4)	0 (0.0)
	Deep venous thrombosis + pulmonary embolism	Asymptomatic, segmental	1 (0.4)	1 (0.7)
		Heavily symptomatic	1 (0.4)	0 (0.0)
	Deep venous thrombosis		3 (1.2)	0 (0.0)
	Visceral thrombosis		2 (0.8)	1 (0.7)
	CVC-related thrombosis		0 (0.0)	0 (0.0)
Bleeding events (symptoms)	Major		1 (0.4)	0 (0.0)
	Minor		1 (0.4)	1 (0.7)

CRC, colorectal cancer; CRP, colorectal polyp; CVC, central venous catheter.

### 3.7 Determinant factors associated with coagulopathy among colorectal cancer patients

In the bivariate logistic regression analysis, old age CRC patients, physical exercise, BMI, tumor size, hypertension, duration since CRC diagnosis, and cancer metastasis were crudely associated with coagulopathy. However, after statistical adjustments in the final model, physical exercise and duration since cancer diagnosis were not independent predictors of the outcome variable (**Table 6**).

**Table 6:** Bivariable and multivariable logistic regression for factors associated with coagulopathy among colorectal cancer patients at referral hospitals of Addis Ababa, Ethiopia, 2022.

Factors	Coagulopathy		95% CI			*p*-value
	Yes, *N* (%)	No, *N* (%)	COR (95% CI)		AOR (95% CI)	
1. Age						
≤50	126	23	1		1	
51–60	31	8	3.71 (1.40, 9.82)		1.91 (1.11, 8.48)	0.74
61–70	29	13	3.61 (0.91, 12.35)		3.13 (1.03, 6.94)	0.001*
>70	12	8	0.96 (0.25, 3.40)		2.73 (1.08, 4.71)	0.001^*^
2. Hypertension						
Yes	63	118	11.7 (9.3, 21.8)		6.8 (1.07, 14.1)	0.03^*^
No	167	74	1		1	
3. Tumor size (cm)						
0–2	19	9	1		1	
2–5	120	16	1.25 (0.65, 2.55)		2.41 (0.59, 3.75)	0.41
5–10	52	25	1.32 (0.64, 3.56)		1.22 (0.81, 3.29)	0.53
≥10	7	2	3.14 (1.43, 6.34)		3.31 (1.11, 6.74)	0.03*
4. Metastasis						
Yes	111	13	15.1 (8.1, 23.6)		5.8 (1.1, 14.7)	.000*
No	87	39	1		1	
5. Body mass index (kg/m2)						
Underweight (<18.5)	68	12	3.1 (0.6, 27.1)		6.7 (0.9, 23.46)	0.098
Normal weight (18.5–24.9)	100	30	1		1	
Overweight (25–29.9)	25	9	4.9 (3.5, 11.4)		6.2 (1.5, 11.4)	.11
Obese (≥30)	5	1	11.5 (1.5, 21.9)		3.8 (2.3, 4.8)	.000*
6. Duration since diagnosed with CRC						
<1.5 years	128	22	1		1	
1.5–3 years	48	14	4.5 (4.7, 9.1)		3.5 (0.4, 9.6)	.22
>3 years	22	16	3.4 (2.4, 6.4)		8.6 (2.6, 16.2)	.11
7. Physical exercise (per week)						
Never	119	34	2.29 (1.2, 3.96)		3.05 (1.03, 4.05)	0.33
Once a week	70	8	7.1 (3.7, 10.4)		6.0 (2.5, 11.8)	0.098
2–3 times	6	8	1.8 (0.2, 16.6)		3.6 (0.2, 52.9)	0.779
4–6 times	3	2	1		1	

The result of the multivariable logistic regression analysis showed that those CRC patients of age between 61 and 70 and greater than 70 years were 3.13 (AOR = 3.13: 95% CI: 1.03, 6.94) and 2.73 (AOR = 2.73, 95% CI: 1.08, 4.71) times more likely to have coagulopathy than younger age groups, respectively. CRC patients who had hypertension were 6.8 (AOR = 6.8; 95% CI: 1.07, 14.1) times more likely to develop coagulopathy than normotensive CRC patients. The likelihood of developing coagulopathy was 3.31 (AOR = 3.31: 95% CI: 1.11, 6.74) times higher in those CRC patients who have a larger tumor size (≥10) than in those with a smaller size. CRC patients who had metastatic cancer were 5.8 (AOR = 5.8; 95% CI: 1.1, 14.7) more likely to develop coagulopathy when compared to those patients who had no metastatic cancer. Finally, cancer patients with a BMI ≥30 kg/m^2^ (obese patients) had 3.8 (AOR = 3.8: 95% CI: 2.3, 4.8) times higher odds of the likelihood of developing coagulopathy than those patients with lower BMI (**Table 6**).

## 4 Discussion

In the present study, thromboembolic events, basic coagulation parameters, and determinant factors associated with the likelihood of developing coagulopathy among CRC patients attending oncology centers in the public referral hospitals of Addis Ababa are reported. Recent evidence has reported that CRC patients are at an increased risk of hemostatic disturbance, causing significant morbidity, worsened quality of life, and poorer prognosis (27–29).

In this study, the overall prevalence of coagulopathy among CRC patients was 198 (79.2%), of whom 190 (76%), 178 (71.2%), and 107 (42.8%) had prolonged PT, INR, and APTT, respectively, while 65 (26%) CRC patients had thrombocytopenia. Comparative analysis also confirmed that the evaluated basic coagulation parameters were significantly higher in CRC patients than in CRP patients and healthy controls (*p* < 0.001). This result was in agreement with the findings reported in Guangzhou, China (27) and two other studies (30, 31) where PT, APTT, and INR were significantly elevated among CRC patients compared with healthy controls (*p* < 0.05). Zhang et al. (27) also discovered that among CRC patients, APTT increased gradually, and PT and APTT can have prognostic value and help predict mortality, as these values increased significantly in patients with advanced-stage CRC than those in an early stage (27). Studies have also revealed that APTT could be used as a reference index, and patients with elevated APTT, especially in the early stage, could have poor prognoses by causing decreases in blood coagulation function, which could lead to colorectal bleeding (32, 33). The extent of elevation of PT, APTT, and INR in this study was higher than that reported in other cancer patients, such as those with breast, multiple myeloma, and lung cancer (34, 35).

Hematologic disturbances among cancer patients are multifactorial, and the mechanisms are not fully understood (36). However, the interaction of cancer cells with the host’s endothelial cells and cancer cell expression of different pro-coagulant molecules, including tissue factor, which has factor-activating properties and induces the formation of platelet microthrombi, are believed to play a role (37–39). Malignant tumor growth, weakened immunity, physical failure, and deranged liver function may also be responsible for elevated serum PT and APTT levels (36).

Patients with CRC are also known to have a substantially higher risk of thromboembolic and bleeding events (40, 41). In the present study, the overall prevalence of thromboembolic events was 17 (6.8%), of which most (9, 52.9%) were in the form of pulmonary embolism. The study result was higher than the results of studies conducted in America (4.1%) (41) and Italy (5.4%) (42) but was lower than a study conducted in the United States (8.4%) (43). A study by Khorana et al. also revealed that thromboembolic events are the main complication in cancer patients, causing death and significantly poorer survival (41). CRP patients were also found to have a risk of thromboembolic and bleeding events, with 6 (4%) patients having thromboembolic events, and bleeding events were observed in 2 (0.8%) CRP patients.

In the analysis of determinant factors that may increase the likelihood of developing coagulopathy among CRC patients, older CRC patients (>60 years) were three times more likely to have coagulopathy than younger age groups. Some studies have asserted that advanced age is a risk factor for coagulopathy and thromboembolism, and these risks further increase among patients with cancer (44). Coagulopathy among elderly cancer patients could be associated with aging of the vascular system, leading to decreased vessel wall integrity and microcirculation, endothelial dysfunction, reduced vascular tone, increased plasma levels of coagulation factors, and sedentary lifestyles, all of which increase the risk of coagulopathy and thromboembolism (45, 46).

Coagulopathy is mostly associated with chronic diseases; in this study, CRC patients with hypertension were significantly associated with coagulopathy. The study participants with hypertension were nearly seven times more likely to develop coagulopathy than those without hypertension. Eleni et al. (2018) reported that hypertensive cancer patients could have exhaustion of the coagulation process, endothelial dysfunction, and chronic activation of the procoagulant pathways, and these patients may also take different medications, which might have an impact on the normal hemostasis process (47).

The likelihood of developing coagulopathy was 6.9 times higher in patients with CRC who had a larger tumor size (≥10 cm) than in those with a smaller tumor size. Similarly, CRC patients who had metastatic cancer were at risk for coagulopathy, as shown by its six times higher odds of association with coagulopathy when compared to patients who had no metastatic cancer. Elevated plasma levels of procoagulant and activated coagulation function have been shown to be associated with angiogenesis, tumor progression, metastasis, and reduced survival in patients with lung cancer (48), gastric cancer (49), renal cell carcinoma (50), and CRC (51). It is hypothesized that tumor cells and host production of procoagulants, fibrinolytic factors, local thrombin, and plasmin are important factors in tumor progression and metastases by enhancing sustained adherence of tumor cells to the vasculature of target organs, supporting vessel formation, and stimulating endothelial cell proliferation during tumor development (48, 49).

Finally, CRC patients with abnormal BMI, especially obesity, had almost four times higher odds of association with coagulopathy than those with a normal BMI. The association between abnormal BMI and coagulopathy, especially in cancer patients, may be related to chronic low-grade inflammation and endothelial dysfunction (52, 53). In addition, obese patients could have increased adipocytokine-mediated activity of coagulation factors, decreased activity of the fibrinolytic system, adipocyte release of tissue factor, increased production of leptin (54, 55) and adiponectin, and production of plasminogen activator inhibitor-1 from adipocytes, all of which result in coagulopathy (56).

## 5 Conclusion and recommendations

Among CRC patients, the burden of coagulopathy has remained a problem of public health importance. To the specific context of the study, older age, hypertension, larger tumor size, metastatic cancer, and obesity were positively associated with coagulopathy. Furthermore, thromboembolic and bleeding events are also significant burdens in patients with CRC. Regular screening is advised due to the growing prevalence of the disease and its complications, especially in those at higher risk, such as those with a personal history of CRC or certain types of polyps; a family history of the condition; a personal history of inflammatory bowel disease, ulcerative colitis, or Crohn’s disease; a confirmed or suspected hereditary CRC syndrome; or a personal history of receiving radiation to the pelvic and abdomen.

The existing efforts in oncology care should be strengthened to prevent coagulopathy and other complications, and cancer care providers should invest in their efforts for early detection using different diagnostic modalities. Moreover, patients with CRPs should be given more emphasis.

## 6 Limitation of the study

Despite being the first in type, the robust methodology we used, and the important findings we reached, the study was not without limitations. The limitations include the small sample size, and due to the high cost of reagents and imaging tests, we have only included 250 colorectal patients and only PT, APTT, INR, and platelet count were measured to assess coagulation profile; the different assays that would help differentiate the exact cause of coagulopathy were not measured. In addition, we did not investigate the impact of the anticancer treatment on the measured parameters.

## Data availability statement

The original contributions presented in the study are included in the article/**Supplementary Material**. Further inquiries can be directed to the corresponding author.

## Ethics statement

The studies involving human participants were reviewed and approved by Ethical Review Board of College of Health Sciences, Addis Ababa University. The patients/participants provided their written informed consent to participate in this study.

## Author contributions

All authors of the study have made a significant contribution to the reported work, which includes the conception, supervision, study design, execution, acquisition of data, statistical analysis, and interpretation of the results, as well as drafting, revising, and critically reviewing the manuscript, and gave final approval of the version to be published. All authors have agreed to the journal to which the article has been submitted and to be accountable for all aspects of the work.
