# Supplementary material for: Evaluation of thromboembolic event, basic coagulation parameters, and associated factors in patients with colorectal cancer: a multicenter study
Source: Front Oncol. 2023 May 2;13:1143122. doi: 10.3389/fonc.2023.1143122 (PMC10188115; doi:10.3389/fonc.2023.1143122)
Supplement: Supplementary file 1 [file Table_1.doc]

STROBE Statement—Checklist of items that should be included in reports of ***cross-sectional studies***

|  | Item No | Recommendation |
| --- | --- | --- |
| **Title and abstract** | 1 | Evaluation of thromboembolic event, Basic Coagulation Parameters, and associated factors in patients with colorectal cancer: A Multicenter Study |
|  |  | Patients with colorectal cancer are at an increased risk of hemostatic disturbances, and recent studies have shown that coagulation disorders could be the first sign of malignancy. Although coagulopathy is a significant cause of cancer-related death and disability, it is usually underestimated, and there has been no recent scientific evidence regarding the exact burden and its specific determinants. An institution-based comparative cross-sectional study was conducted on a total of 500 study participants (250 colorectal cancer, 150 colorectal polyp, and 100 control groups) from January to December 2022. The study result showed that, the prevalence of coagulopathy among colorectal cancer patients was 198 (79.2%; 95% CI: 73.86, 83.64), while the prevalence was 76 (50.7%; 95% CI: 45.66, 54.34) among colorectal polyp patients. From the final model, age between 61-70 years and, age > 70 years, hypertension, larger tumour size, metastatic cancer, and BMI ≥30kg/m2 were positively associated with coagulopathy. |
| Introduction | | |
| Background/rationale | 2 | Acute and chronic cancer-related complications that arise either as an initial malignancy manifestation or due to its progression are often the main cause of cancer-related mortality and hospital admission. These complications influence the prognosis and timing of cancer diagnosis, as well as the timing and receipt of treatment outcomes. Cancer patients are usually associated with an increased risk of hemostatic disturbances and thromboembolic events. To avoid cancer-related complications, such as coagulopathy, minimize mortality, and enhance patient quality of life, it is critical to recognize and manage risk factors early. In Ethiopia, despite the increased incidence and prevalence of CRC and its complications, relatively high incidence of CRC mortality, and high rate of delay to diagnosis, CRC receives a relatively low public health priority due to low public awareness, scarce financial resources, weak health care systems, a shortage of oncologists, and an already overburdened economy. However, data on the prevalence and determinants of coagulopathy among patients with CRC in Ethiopia are limited. |
| Objectives | 3 | To evaluate the prevalence of coagulopathy among colorectal cancer patients  To evaluate the prevalence of thromboembolic event among colorectal cancer patients  To assess the prevalence of bleeding event among colorectal cancer patients  To Determinant factors associated with coagulopathy among colorectal cancer patients |
| Methods | | |
| Study design | 4 | An institution-based comparative cross-sectional study was conducted from January to December 2022 at oncology centers in referral hospitals of Addis Ababa, Ethiopia. |
| Setting | 5 | Starting from January 1, 2022, all consecutive patients (colorectal cancer and colorectal polyp) were evaluated on the first day of diagnosis and/or hospitalization, and those fulfilling the eligibility criteria were included. |
| Participants | 6 | All adult colorectal cancer and colorectal polyp patients whose age was greater than 18 years and visiting the selected hospitals during the study period (from January 2022 to December 2022) were included in the study. |
| Variables | 7 | Outcomes; coagulopathy, thromboembolic event, and bleeding event.  Predictors; patient-related (advanced age, race, sex, comorbidities, obesity, history of thrombosis), cancer-related (primary tumor site, disease stage/grade, cancer histology type, duration since initial diagnosis), and cancer treatment-related (chemotherapy, radiotherapy, surgery, anti-angiogenic agents, hormonal therapy, and transfusions) factors are associated with an increased risk of developing coagulopathy,  Diagnostic criteria; Coagulopathy was considered an abnormality in one of the basic coagulation parameters assessed: prolonged PT and/or APTT values, thrombocytopenia, abnormally high PT/international normalized ratio (INR), or APTT).  Normal time for PT: The normal time for PT was considered between 10 and 14 s.  Normal time for APTT: The normal APTT time was considered 24–36 seconds  Abnormal high INR: The normal INR was considered 0.8–1.2  Normal platelet count: Platelet count between 150,000 and 400,000/µL |
| Data sources/ measurement | 8* | Both primary and secondary data were collected. Primary data, blood sample, secondary data were abstracted from patient’s medical chart by medical record review using a structured checklist with physical examination. |
| Bias | 9 | All methods were used to address potential sources of bias |
| Study size | 10 | We don’t use sample size calculation formula as the prevalence of coagulopathy among CRC patients is not known and because of economic constraint we only include all patients visiting the selected hospitals during the study period and add controls proportional to the included patients. |
| Quantitative variables | 11 | Percentages mean, median, IQR, and standard deviations, were used to present quantitative variables and were handled appropriately in the analyses. |
| Statistical methods | 12 | (*a*) Binary logistic regression analysis was used to examine independent variables associated with coagulopathy. |
|  |  | (*b*) Non-parametric Kruskal–Wallis tests followed by Dunn-Bonferroni pairwise comparison tests were used to compare the median (IQR) values of the different serum parameters between the case and control groups. |
|  |  | (*c*) There were no missing data |
|  |  | (*d*) mentioned above |
|  |  | (*e*) no sensitivity analyses |
| Results | | |
| Participants | 13* | (a) The study participants were divided into three groups: Group I comprised 250 histopathologically confirmed CRC patients, diagnosed and on chronic follow-up; CRC patients who were admitted for anti-cancer treatment (chemotherapy/surgery); and CRC patients admitted due to any cancer related complication management but who did not receive any type of anticancer treatment at the selected hospitals during the data collection period. To analyse and compare the evaluated basic coagulation parameters, in addition to CRC patients, our study also recruited 150 histopathologically confirmed (colonoscopy or flexible sigmoidoscopy) colorectal polyp patients who visited the selected hospitals or were on chronic follow-ups, and who did not receive any type of treatment during the data collection period as group II and 100 apparently healthy volunteers as the third group (group III), which included apparently healthy adults who visited the selected study areas for any reason (clinical and administrative staff members, patient attendants) during the study period. |
|  |  | (b) we had 100percente response rate |
|  |  | (c) not applicable |
| Descriptive data | 14* | (a) Socio-demographic characteristics and related information, an interviewer-based pretested and structured questionnaire was used. Clinical and histopathological data (medical conditions/comorbidities, HIV/AIDS status, primary tumor location (colon, rectal), tumor size, sites of metastases, risk factors for coagulopathy, and BMI) |
|  |  | (b) there were no participants with missing data for any variable |
| Outcome data | 15* | The outcome variables were coagulopathy, thromboembolic event, and bleeding tendency. |
| Main results | 16 | (*a*) no confounder was considered for this particular study |
|  |  | (*b*) continuous variables were expressed in round of two and were categorized by using internationally agreed cut of points |
|  |  | (*c*) not applicable |
| Other analyses | 17 | All statistical analyses were mentioned above. |
| Discussion | | |
| Key results | 18 | The prevalence of coagulopathy among colorectal cancer patients was 198 (79.2%; 95% CI: 73.86, 83.64), while the prevalence was 76 (50.7%; 95% CI: 45.66, 54.34) among colorectal polyp patients. From the final model, age between 61-70(AOR= 3.13: 95% CI: 1.03, 6.94), age > 70 years (AOR= 2.73: 95% CI: 1.08, 4.71), hypertension (AOR= 6.8: 95% CI: 1.07, 14.1), larger tumor size (AOR= 3.31: 95% CI: 1.11, 6.74), metastatic cancer (AOR= 5.8: 95% CI: 1.1, 14.7), and BMI ≥30kg/m2 (AOR= 3.8: 95% CI: 2.3, 4.8) were positively associated with coagulopathy. |
| Limitations | 19 | The limitations include small sample size and due to high cost of reagents and imaging tests we have only included 250 colorectal patients and only PT, APTT, INR, and platelet count were measured to assess coagulation profile and the different assays that would help differentiating the exact cause coagulopathy were not measured. In addition, we did not investigate the impact of the anticancer treatment on the measured parameters. |
| Interpretation | 20 | The interpretation of the study was in line with objectives considered and interpretations of results were made. Most of the study results were above most other international studies. |
| Generalisability | 21 | As the study was conducted by including all colorectal cancer patients in almost all hospitals, generalization is possible. |
| Other information | | |
| Funding | 22 | No funding was received for this particular study |

*Give information separately for exposed and unexposed groups.

**Note:** An Explanation and Elaboration article discusses each checklist item and gives methodological background and published examples of transparent reporting. The STROBE checklist is best used in conjunction with this article (freely available on the Web sites of PLoS Medicine at http://www.plosmedicine.org/, Annals of Internal Medicine at http://www.annals.org/, and Epidemiology at http://www.epidem.com/). Information on the STROBE Initiative is available at www.strobe-statement.org.
